# Selective serotonin reuptake inhibitors for amblyopia treatment: a systematic review and meta-analysis of randomized controlled trials

**DOI:** 10.3389/fneur.2025.1639913

**Published:** 2025-08-21

**Authors:** Ehtesham Shamsher, Luisa Saccaro, Victor Barreiros Pungirum, Nghi Bao Tran, Omar A. H. A. Alghaith, Fatemeh Khabazianzadeh, Holly Bridge, I. Betina Ip

**Affiliations:** ^1^Oxford Eye Hospital, John Radcliffe Hospital, Oxford University Hospitals NHS Foundation Trust, Oxford, United Kingdom; ^2^Faculty of Medicine, Universidade de Mogi das Cruzes, Mogi das Cruzes, Brazil; ^3^Faculty of Medical Sciences, University of Buenos Aires, Buenos Aires, Argentina; ^4^Faculty of Medicine, University of Debrecen, Debrecen, Hungary; ^5^Institute of Ophthalmology, University College London, London, United Kingdom; ^6^Eye Research Center, Khatam-al-Anbia Hospital, Mashad University of Medical Sciences, Mashhad, Iran; ^7^Nuffield Department of Clinical Neurosciences, Oxford University Centre For Integrative Neuroimaging, Oxford, United Kingdom

**Keywords:** amblyopia, strabismus, anisometropia, fluoxetine, citalopram, SSRIs, neuroplasticity

## Abstract

**Introduction:**

Amblyopia is a neurodevelopmental visual disorder treated with occlusion or pharmacological penalization of the dominant, non-amblyopic eye in early childhood. After early childhood, efficacy of occlusion therapy is limited due to a reduction in neuronal plasticity, and no mainstay clinical treatment is available. Selective serotonin reuptake inhibitors (SSRIs) have been hypothesized to enhance neuroplasticity in the adult brain, thereby facilitating improvements in amblyopia. We aimed to perform a systematic review and meta-analysis evaluating the effect of SSRIs on patients with amblyopia.

**Methods:**

We systematically searched Pubmed, EMBASE, and Cochrane Central for randomized controlled trials (RCTs) and controlled observational studies comparing an SSRI with placebo in patients with amblyopia. Outcomes of interest were visual acuity (VA) change and visual evoked potential (VEP) change (P100 amplitude and latency). Statistical analysis was performed using the web version of RevMan by calculating the mean difference between groups (MD). Heterogeneity was assessed with Cochrane Q test and I^2^ statistics.

**Results:**

Four RCTs and 139 patients were included. 55% patients received SSRIs. Three studies used fluoxetine and one study used citalopram as the intervention. While SSRIs use statistically improved VA (MD 0.09 logMAR, 95% CI 0.04–0.14, *p* = 0.0004), the extent of improvement was not clinically significant. SSRIs did not have any effect on VEPs.

**Conclusion:**

While SSRIs significantly improved VA in patients, the increase was not clinically significant as it represents less than one line of improvement on the Snellen chart. Given the minimal change in VA, it may be necessary to combine SSRIs with other modalities of intervention to demonstrate a clinically significant effect. Secondary endpoints that capture effects at the level of the retina and the brain would provide knowledge of physiological mechanisms that can improve future therapies.

**Systematic review registration:**

CRD42025633077, https://www.crd.york.ac.uk/PROSPERO/view/CRD42025633077.

## Introduction

Amblyopia is a neurodevelopmental visual disorder characterized by reduced visual acuity in one eye with a prevalence reported to be between 1 and 5% in children (1, 2). Abnormal visual input during early childhood, a critical period of visual development, can lead to unequal quality of retinal stimulation between the two eyes. Specifically, the neural connections from the affected eye do not develop normally, leading to a disruption in the balance of input from the two eyes in the visual cortex and suppression of input from the amblyopic eye (3–5). Prolonged and asymmetric suppression may lead to neural defects in the representation of the deviating eye, potentially causing amblyopia and favoring the dominant eye (5). Common causes are anisometropia (difference in refraction power between both eyes of at least +1.00), strabismus (misalignment of the eyes causing eso-, exo-, hypo- or hypertropia), or deprivation (e.g., congenital cataracts, ptosis) (6). If untreated during childhood, amblyopia can result in permanent loss of normal monocular vision and impaired binocular function (6).

The standard therapeutic intervention for amblyopia commences with refractive adaption which allows the visual acuity deficits to be fully corrected in 25% children (7). If it is not corrected, occlusion of the dominant, non-amblyopic eye using a patch or pharmacological penalization, such as atropine drops, to stimulate the amblyopic eye can be performed (6). This approach leverages neural plasticity to increase the strength of visual input from the weaker eye. However, the effectiveness of this treatment is age-dependent and attempted early around age 5, with diminishing returns observed as the critical period for visual cortical plasticity closes around 8 years old (8). In adults, the efficacy of occlusion therapy is thought to be markedly limited due to reduced synaptic remodeling capacity (6, 9). Therefore, novel treatment paradigms are needed to treat amblyopia in adults.

Selective serotonin reuptake inhibitors (SSRIs) such as fluoxetine and citalopram have been hypothesized to enhance neuroplasticity in the adult brain, offering a potential pharmacological treatment for amblyopia in adults when used in combination with vision therapy (10). Fluoxetine, in particular, has shown promise in preclinical studies, where it has been demonstrated to reactivate ocular dominance plasticity in animal models by increasing the brain-derived neurotrophic factor (BDNF) protein expression and decreasing GABAergic inhibition in the visual cortex (11). This suggests that SSRIs might open a therapeutic window for visual recovery beyond the critical period. However, the evidence from trials has been inconsistent, with some studies reporting significant improvements in visual outcomes (12, 13), while others fail to demonstrate efficacy (9, 14). Given the contradictory findings in the published literature and the absence of guidelines on the use of SSRIs in amblyopia, we performed a systematic review and meta-analysis to synthesize existing data and assess the overall efficacy of SSRIs in treating amblyopia to give both clinicians and basic scientists more accurate information.

## Methods

### Search strategy and eligibility criteria

This systematic review and meta-analysis was conducted and documented following the guidelines outlined in the Cochrane Collaboration Handbook for Systematic Reviews of Interventions and the Preferred Reporting Items for Systematic Reviews and Meta-Analyses (PRISMA) Statement (15). The research protocol was published using Prospero (registration no. CRD42025633077). Two authors independently systematically searched Pubmed, Embase and Cochrane from inception to January 7, 2025 for studies published in English using the following search query: (amblyopia OR anisometropic OR anisometropia OR strabismic OR strabismus OR lazy) AND (fluoxetine OR citalopram OR escitalopram OR dapoxetine OR fluvoxamine OR paroxetine OR sertraline OR vorioxetine OR SSRI OR SSRIs OR serotonin). The same search query was used for each database. Additionally, the references of the included studies were examined to identify any additional relevant studies. Disagreements were resolved by consensus.

We included studies that met the following criteria: (1) randomized controlled trials (RCTs), (2) comparing any SSRI with placebo in patients with amblyopia, (3) reporting any outcome of interest. We excluded the following studies: (1) case reports, editorial letters, reviews, or (2) studies with missing data on the interventional group or control group or overlapping patient populations.

### Data extraction

Data extraction was performed independently by two authors based on predefined search criteria. Data were gathered from tables, text, and graphs. Any disagreements were resolved through consensus.

### Endpoints

The primary endpoint was visual acuity (VA) improvement after SSRI treatment. VA was measured using the logarithm of the minimum angle of resolution (logMAR) chart. If the endpoint was not presented as a difference from baseline, it was calculated using the data available. The secondary endpoint was pattern-reversal visual evoked potential (VEP) change (P100 amplitude and latency), an electrophysiological metric that evaluates cortical responses to visual stimulation.

### Quality assessment (risk of bias)

The risk of bias assessment was conducted by two independent authors using the Cochrane Collaboration’s tool for evaluating the risk of bias in randomized trials (RoB2) (16). Each study was rated as having a high, low, or unclear risk of bias across five domains: selection bias, performance bias, detection bias, attrition bias, and reporting bias. To evaluate potential publication bias, funnel plots comparing study weights to point estimates were analyzed (17). Discrepancies were resolved through consensus following a discussion of the reasons for the differences.

### Statistical analysis

Statistical analysis was performed using Review Manager. Continuous outcomes were pooled using mean differences (MD). For crossover studies, a paired student *T*-test was used followed by a generic inverse-variance method to pool them with non-crossover RCTs. Heterogeneity was assessed using the Cochrane Q test and I^2^ statistics, with *p* < 0.10 and I^2^ > 25% considered indicative of significant heterogeneity. A random-effect model was applied. A leave-one-out sensitivity analysis was carried out by removing the crossover RCT to ensure the results were not dependent on it.

## Results

**Figure 1:**
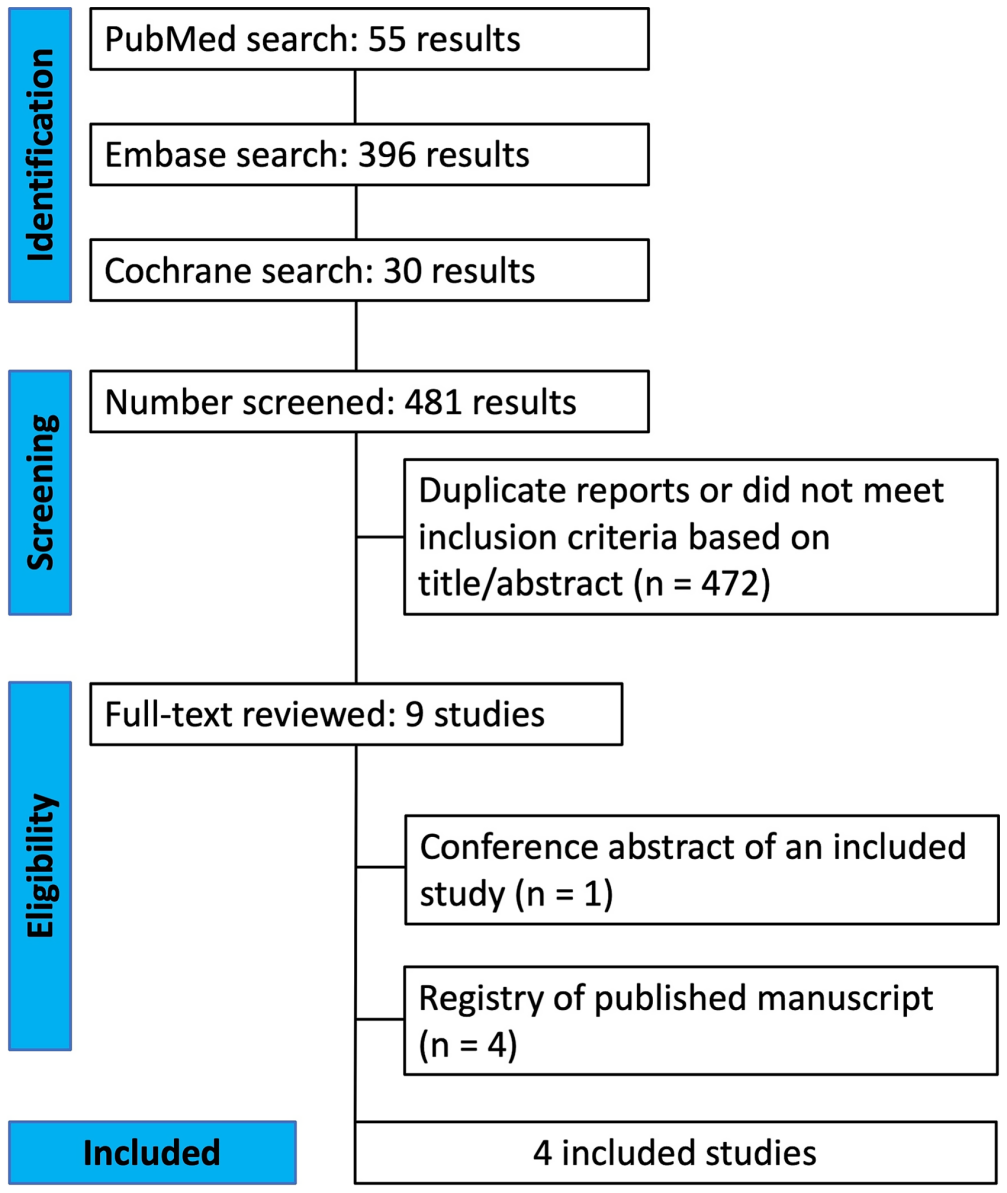
PRISMA flow diagram of study screening and selection.

The initial search identified 481 results. After excluding duplicates and studies that did not meet the eligibility criteria, nine studies were assessed in detail based on the inclusion criteria. Four studies (9, 12–14) were included, encompassing 139 patients from four RCTs (Figure 1). Among these, a total of 76 patients (55%) received SSRIs including five patients from a crossover study. All patients had their dominant eye occluded daily between 1 and 6 h. The age range of included patients was between 11 and 57 years old. Study characteristics are reported in Table 1. Three studies had fluoxetine and one citalopram as the intervention group.

**Table 1:** Baseline characteristics of included studies.

Study	Design	Patients SSRI/Pl	Intervention (SSRI)	Length of patching, concomitant training	Male, % SSRI/Pl	Age^†^, y SSRI/Pl	Age range, y SSRI/Pl	Type of amblyopia, %: anisometropic, strabismic, combined SSRI/Pl	Baseline VA^§^, logMAR SSRI/Pl	Follow-up
Huttunen et al. (9)	RCT	22/20	20 mg fluoxetine	1 h/day, game-basedperceptual training	50/55	38.5 ± 12.5/36.4 ± 11.5	20–57/19–57	82/95 4/0 14/5	0.649 ± 0.252/0.620 ± 0.190	10 weeks
Lagas et al. (14)	RCT^*^	5/2	20 mg citalopram	2 h/day, perceptual training	80/50	28.8 ± 10.4/45 ± 2.8	19–44/43–47	20/50 40/0 40/50	0.596 ± 0.476/0.82 ± 0.25	2 weeks
Sharif et al. (12)	RCT	20/15	0.5 mg/kg fluoxetine	4-6 h/day, no	40/53.3	21 ± 8/21 ± 7	11–37/12–35	NA	0.490 ± 0.148/0.493 ± 0.138	3 months
Mirmohammadsadeghi et al. (13)	RCT	29/26	20 mg fluoxetine	4 h/day, no 2 h/day,	58.6/61.5	25.9 ± 8.9/28.8 ± 8.1	18–54/18–46	41.4/30.8 41.4/30.8 17.2/38.4	0.55 ± 0.29/0.51 ± 0.31	3 months

^†^Mean ± SD; ^§^amblyopic eye with mean ± SD; *cross-over design with data presented with first treatment; SSRI, selective serotonin recapture inhibitor; Pl, placebo; NA, not available; RCT, randomized controlled trial; y: year.

**Figure 2:**
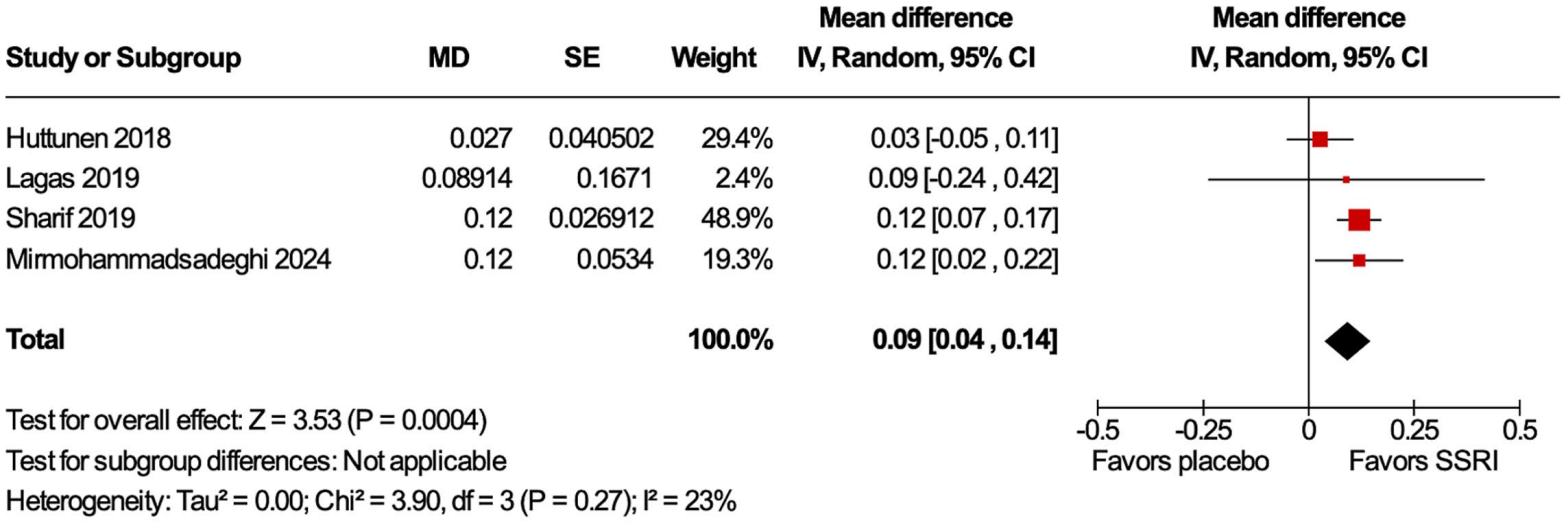
Visual acuity (VA) improved with selective serotonin receptor inhibitor (SSRI). VA improvement is statistically significant in the SSRI group compared to placebo.

**Figure 3:**
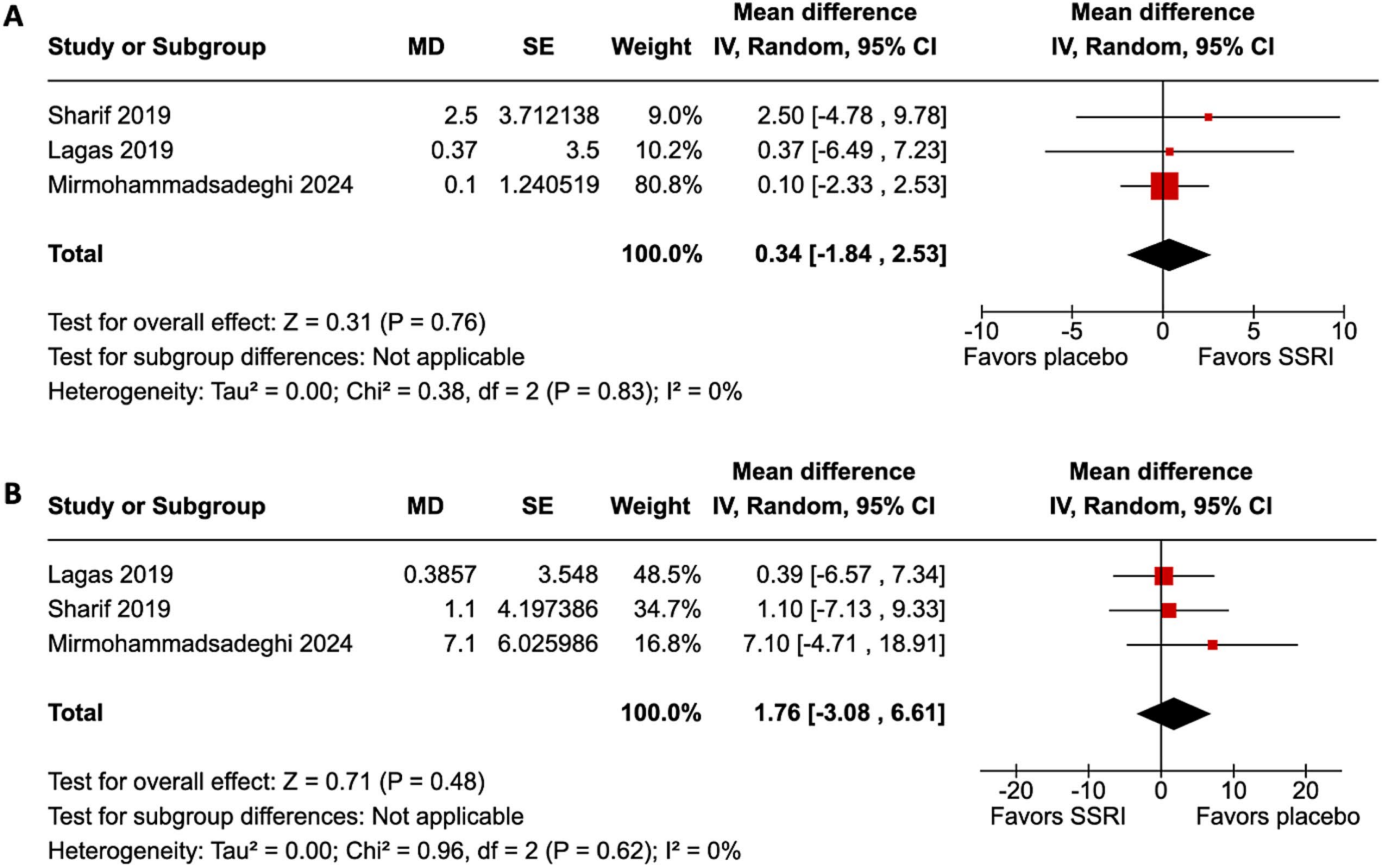
Visual evoked potential (VEP) did not improve with SSRI. **(A)** P100 amplitude improvement was not statistically significantly different between the SSRI and placebo group. **(B)** P100 latency improvement was not statistically significantly different between the SSRI and placebo group.

All four studies (9, 12–14) included the primary endpoint. Patients in all studies had their dominant eye patched. Patients treated with an SSRI had an improvement in VA compared to placebo (MD 0.09 logMAR, 95% confidence interval [CI] 0.04–0.14, *p* = 0.0004, I^2^ = 23%, Figure 2). Only three studies (12–14) included the secondary endpoint. SSRIs did not change VEP compared to placebo. There were no differences in P100 amplitude (MD 0.34 μV, 95% CI −1.84–2.53, *p* = 0.76, I^2^ = 0%, Figure 3A) or P100 latency (MD 1.76 ms, 95% CI −3.08–6.61, *p* = 0.48, I^2^ = 0%, Figure 3B) between intervention and placebo groups. The sensitivity analysis for both outcomes did not change the effect size.

**Figure 4:**
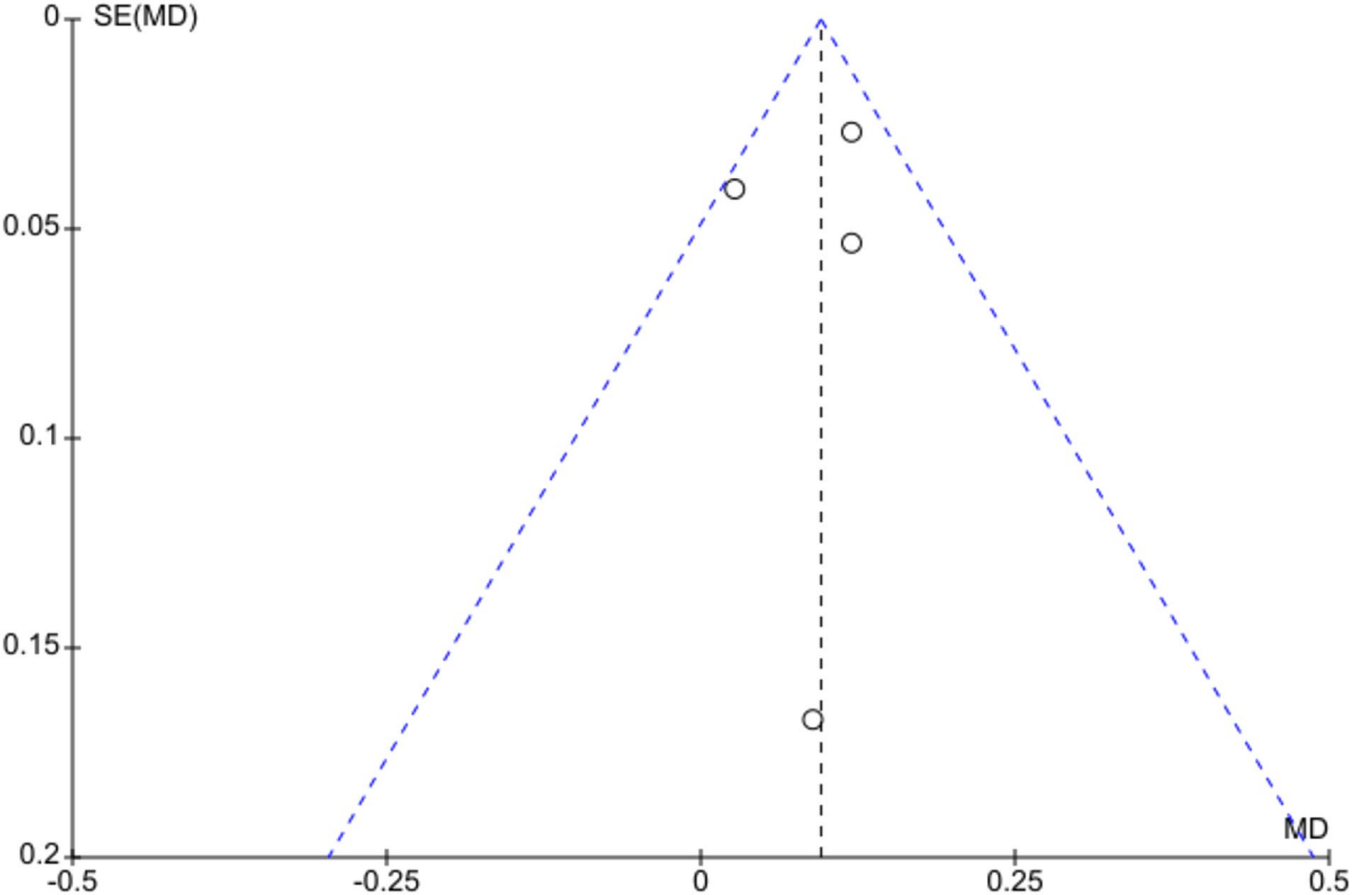
Funnel plot for visual acuity improvement. There is no publication bias.

All studies were evaluated as having a low risk of bias (Table 2). Funnel plot analysis revealed no evidence of publication bias. The studies with comparable weights and MD were symmetrically distributed, except for the crossover trial (Figure 4).

**Table 2:** Risk of bias summary for randomized studies.

Study	Bias from randomization process	Bias due to deviations from intended interventions	Bias due to missing outcome data	Bias in measurement of the outcomes	Bias in selection of the reported result	Overall risk of bias
Huttunen et al. (9)	Low	Low	Low	Low	Low	Low
Lagas et al. (14)	Low	Low	Low	Low	Low	Low
Sharif et al. (12)	Low	Low	Low	Low	Low	Low
Mirmohammadsadeghi et al. (13)	Low	Low	Low	Low	Low	Low

## Discussion

This systematic review and meta-analysis of four studies with a total of 139 patients compared the use of an SSRI (fluoxetine and citalopram) against placebo for amblyopia. While SSRIs slightly improved VA, the improvement was not clinically significant. Moreover, it did not change either VEP amplitude or latency.

Current evidence on the use of SSRIs for amblyopia is lacking as each clinical trial reports contradicting results with a low number of participants (9, 12–14) so, given the lack of treatment for amblyopia in adults, this meta-analysis pooled the available data on this topic.

SSRIs are used as a first-line pharmacotherapy for depression in both adult and pediatric patients. The therapeutic mechanism is inhibition of the serotonin transporter (SERT) at the presynaptic terminal, thereby increasing the availability of serotonin in the synaptic gap and potentiating postsynaptic receptors stimulation (18). This mechanism not only addresses the monoamine hypothesis of depression but also promotes neuroplasticity and cortical activation, making SSRIs a potential adjuvant therapy for conditions such as amblyopia (19). They are well tolerated but risks such as QT interval prolongation and increased suicidality require careful monitoring. These properties highlight SSRIs as a promising option for improving visual outcomes when used in conjunction with occlusion of the dominant eye (19).

In addition to the serotonergic system, dopaminergic and cholinergic systems have been advocated to play a role in the treatment of amblyopia. Levodopa was tested as an adjunct treatment with occlusion of the dominant eye in a randomized clinical trial including 7 to 12 years old children with residual amblyopia but did not show any benefit (20). Donepezil showed an improvement of the VA in an open-label pilot study (21).

Across the studies, there was a statistically significant improvement of VA with SSRI treatments compared to placebo. This finding is consistent with preclinical studies suggesting that SSRIs may enhance neuroplasticity in the visual cortex of rats and reactivate eye dominance after critical periods (11). In previous clinical trials, the results were contradictory as some studies showed benefit of SSRI treatment (12, 13) whereas others did not (9, 14). Although in this meta-analysis the VA improvement was statistically significant with SSRI treatments, it was not clinically significant as this represents an improvement of less than one line on the Snellen VA chart. Although amblyopia has been studied on animal models for decades, animal models do not necessarily reflect the complexity of the human visual pathway. Rodent models are mostly used for deprivation amblyopia and the magnitude of improvements is less than in humans (22). For example, monocular deprivation only leads to a reduction of a single octave in grating acuity while a significant decrease in visual acuity would be expected in humans (22). Similarly, measuring of visual acuity in rodents and humans cannot be standardized (22). Mitchell et al. (22) recommends using the “two-species rule” before starting any clinical trials to overcome the limitations of a single animal model and increase the likelihood that an intervention will translate to humans (22). VEPs were not significantly changed with SSRIs, suggesting that the VA improvement was not translated into electrophysiological changes. This is consistent with previous clinical studies (12, 13) and might suggest that the VA improvement is not sufficient to impact neuronal plasticity or, more likely, VEPs are not sensitive enough to detect these small changes. As the pattern-reversal VEP primarily assesses the integrity of the central visual pathway and represents a summed cortical signal, it may not capture small or spatially restricted changes (23). There is a need for better endpoints that capture neural processes underlying amblyopia. Such endpoints should reflect physiological mechanisms, providing knowledge that can be used to improve therapies. Other techniques may be more sensitive to small changes in neuronal plasticity related to amblyopia such as magnetic resonance spectroscopy (MRS) (24) which measures concentrations of neurochemicals including GABA and glutamate and could be used in future clinical trials (25–27). MRS data can be collected in combination with established clinical endpoints such as best-corrected visual acuity in clinical trials to evaluate changes in the brain regions underlying amblyopic vision. Promisingly, changes in eye dominance in normally sighted relate to MRS-measured GABA (28), indicating that the technique could also be sensitive to neuroplasticity due to therapy in amblyopes.

Given the wide availability in the clinic and ease of data collection, Optical Coherence Tomography (OCT) would be optimally placed as an additional end point. However, while thinning in the macula has been reported in both fellow and amblyopic eyes after occlusion therapy (29), the magnitude of changes were not correlated to individual improvement. Moreover, the longitudinal arm of this study was very small and the occlusion around 12 months. Patching performed over weeks, would unlikely result in structural changes in structural changes. The utility of OCT for assessing neural changes following short term therapies has yet to be proven.

Heterogeneity between studies included in this meta-analysis was minimal for both endpoints, indicating consistent effect sizes across clinical trials. The sensitivity analysis did not show a different effect size when removing the clinical trial with citalopram suggesting that fluoxetine and citalopram had the same effect. Risk of bias assessment showed that all included studies had a low risk of bias and there was no publication bias, strengthening the reliability of our findings.

To our knowledge, this is the first meta-analysis showing an effect of SSRIs for the treatment of amblyopia in combination with occlusion of the dominant eye. Since this study only includes RCTs, it is a robust synthesis of all the available evidence in the literature.

Other modalities of treatment are also currently under research. Extended-reality (XR) devices (30), physical activity (cycling) (31) and more recently the occlusion of the amblyopic eye combined with physical activity (32) showed some improvement in the adult amblyopic eye. It may be that including SSRI treatment alongside these interventions may increase any effects.

In terms of limitations, the number of patients included is low because there are only four published clinical trials, each of which has a small number of patients. Secondly, one of the four studies included is a crossover trial (16) which leads to non-independence of effect size. This can also lead to a possible carryover effect, but it is reduced by having a washout period of 2 weeks, allowing citalopram to be completely washed out. However, a sensitivity analysis did not show any significant difference when this crossover trial was excluded. Thirdly, the patients included had anisometropic, strabismic or combined amblyopia, limiting the generalization of our results to other types of amblyopia. In addition, the age of patients included was between 11 and 57 years old. Given the variability in plasticity across this age range, a further sensitivity analysis was performed that showed no significant difference when the study that included patients under 18 years old was removed. It would be helpful to determine the potential interaction between occlusion therapy and SSRIs, and how much of the effect can be attributed the SSRI alone compared to combined treatment. Unfortunately, no clinical trials have investigated the effect of SSRIs alone on amblyopia without patching. Finally, no data were included on the safety profile of SSRIs in these clinical trials, making it difficult to assess the tradeoff between therapeutic effects and risks.

## Conclusion

This meta-analysis, which comprises 139 patients, shows that SSRIs slightly improve VA in patients with amblyopia when combined with the occlusion of the dominant eye. However, while larger trials may increase the statistical power and inform of a more representative and generalisable effect, the current evidence shows that the limited improvement does not translate to clinically significant results. There is a need for endpoints to capture changes at the level of the retina and the brain that may relate to perceptual changes.

## Data Availability

The original contributions presented in the study are included in the article, further inquiries can be directed to the corresponding author.
